# The *Yersinia enterocolitica *type three secretion chaperone SycO is integrated into the Yop regulatory network and binds to the Yop secretion protein YscM1

**DOI:** 10.1186/1471-2180-7-67

**Published:** 2007-07-05

**Authors:** Svea Dittmann, Annika Schmid, Susanna Richter, Konrad Trülzsch, Jürgen Heesemann, Gottfried Wilharm

**Affiliations:** 1 Max von Pettenkofer-Institut, Lehrstuhl für Bakteriologie, Ludwig-Maximilians-Universität München, Pettenkoferstr. 9a, D-80336 München, Germany; 2 Robert Koch-Institut, Bereich Wernigerode, Burgstr. 37, D-38855 Wernigerode, Germany

## Abstract

**Background:**

Pathogenic yersiniae (*Y. pestis*, *Y. pseudotuberculosis*, *Y. enterocolitica*) share a virulence plasmid encoding a type three secretion system (T3SS). This T3SS comprises more than 40 constituents. Among these are the transport substrates called Yops (*Yersinia *outer proteins), the specific Yop chaperones (Sycs), and the Ysc (Yop secretion) proteins which form the transport machinery. The effectors YopO and YopP are encoded on an operon together with SycO, the chaperone of YopO. The characterization of SycO is the focus of this study.

**Results:**

We have established the large-scale production of recombinant SycO in its outright form. We confirm that *Y. enterocolitica *SycO forms homodimers which is typical for Syc chaperones. SycO overproduction in *Y. enterocolitica *decreases secretion of Yops into the culture supernatant suggesting a regulatory role of SycO in type III secretion. We demonstrate that *in vitro *SycO interacts with YscM1, a negative regulator of Yop expression in *Y. enterocolitica*. However, the SycO overproduction phenotype was not mediated by YscM1, YscM2, YopO or YopP as revealed by analysis of isogenic deletion mutants.

**Conclusion:**

We present evidence that SycO is integrated into the regulatory network of the *Yersinia *T3SS. Our picture of the *Yersinia *T3SS interactome is supplemented by identification of the SycO/YscM1 interaction. Further, our results suggest that at least one additional interaction partner of SycO has to be identified.

## Background

An ever increasing number of Gram-negative bacteria is known to utilize a type three secretion system (T3SS), mostly pathogens that manipulate their host cells by injecting effector proteins [1]. One of the characteristics of T3SSs is the existence of specific energy-independent chaperones which assist several of the transport substrates. Chaperones assisting the effectors (class I chaperones) are distinguished from those assisting the pore forming translocators (class II chaperones) [2]. The mode of interaction between class I chaperones and their respective substrates is well-investigated, mainly due to crystal structures of several chaperone/substrate complexes [3-5]. Typically, a region of 50–80 amino acids following the putative N-terminal secretion signal is wrapped around a chaperone dimer thereby preventing tertiary structure formation of this substrate region. The rest of the substrate, however, is folded [4-8]. The chaperone/substrate complexes are recognized by a T3SS-specific ATPase [9], which is able to displace the chaperone from its substrate and further to act as an unfoldase unraveling the substrate [10].

Despite these recent discoveries, the substrate recognition process has remained enigmatic [11,12]. It has been suggested that the chaperone/substrate complexes may constitute a three-dimensional secretion signal [4]. This hypothesis is supported by the intriguing fact that the structures of all classI chaperones share a common fold even across the genus barrier and despite low sequence similarity.

The T3SS of pathogenic *Yersinia spp*. is among the most intensively studied T3SSs with the structures of the homodimeric chaperones SycE [13-15], SycH [16], SycT [17,18], and the atypical SycN/YscB heterodimer [5] being solved. According to phylogenetic analyses and experimental results, the *Yersinia *T3SS holds at least one additional classI chaperone, SycO [19,20].

Here, we focus on characterization of *Y. enterocolitica *SycO, encoded by a gene termed *orf155 *until recently. In *Y. enterocolitica*, *sycO *is organized in an operon together with *yopO *(*ypkA *in *Y. pseudotuberculosis *and *Y. pestis*) and *yopP *(*yopJ *in *Y. pseudotuberculosis *and *Y. pestis*). Within this operon, *sycO *is located upstream of the *yopO *gene. A nonpolar deletion mutant of *orf1*, the *sycO *orthologue in *Y. pseudotuberculosis*, secreted YpkA and YopJ in amounts comparable to the wildtype and was fully virulent. Further, *orf1 *was not found to be involved in temperature and calcium-dependent control of Yop expression and secretion [21]. Because of these results and the use of the infrequent "GTG" start codon of *orf1*, Galyov *et al*. [21] proposed that *orf1 *might not be translated. Later, the corresponding *orf7 *of *Y. pestis *was discussed as a potential Syc-like chaperone due to the typical size (15.7 kDa), isoelectric point (pI of 4.39), and amphipathic character of the putative translation product [22]. However, in *Y. enterocolitica *the induction of apoptosis by YopP in macrophages was shown to be independent of *orf155*, and it was found that secretion of YopO into the culture supernatant was apparently not restricted in the absence of *orf155 *[23]. Only recently, a chaperone function of the *orf155 *product on the effector YopO was reported. Consequently, the gene product of *orf155 *was termed SycO and the gene was renamed *sycO *[20].

Here, we characterize recombinantly produced SycO as a homodimer. We demonstrate the potential of SycO to influence Yop expression and secretion, and identify a novel interaction partner of SycO.

## Results

### Recombinant production of SycO

To examine SycO *in vitro*, we recombinantly produced SycO in *E. coli *and purified it to near homogeneity. For this purpose, we followed a protocol previously established for production of SycH and SycT to generate the proteins in their outright form without an affinity tag [7,18]. The *sycO *sequence of *Y. enterocolitica *strain WA-314 [24] was amplified by PCR, thereby changing the start codon from "GTG" to "ATG" to ensure maximal expression. The fragment was cloned into vector pWS [18]. Expression was performed in *E. coli *BL21 (DE3) pLysS. SycO was produced in abundance and its solubility was optimized by expression at 27°C overnight. A purification protocol was established combining ammonium sulfate precipitation followed by MonoQ-based anion exchange chromatography and a final gel filtration step. The yield was approximately 15 mg of purified protein per liter of culture with a homogeneity of about 95%.

### Recombinant SycO forms homodimers

**Figure 1:**
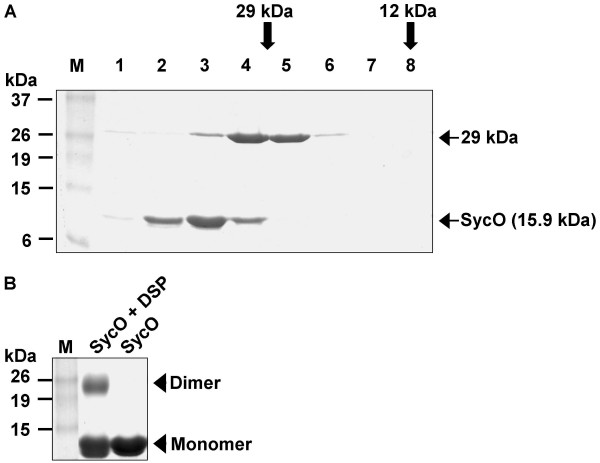
Recombinant SycO forms homodimers. (A) Analytical gel filtration shows that SycO elutes from a Superdex 75 column prior to the 29 kDa marker protein, carbonic anhydrase. Consecutive fractions were analyzed by SDS-PAGE (Coomassie-stained); the column was calibrated as indicated above the gel. (B) Chemical cross-linking of recombinant SycO using DSP as cross-linking reagent was analyzed by Coomassie-stained SDS-PAGE.

Typically, T3SS effector chaperones form homodimers. In order to test SycO for oligomerization, we applied analytical gel filtration and chemical cross-linking. Fig. 1A depicts the SDS-PAGE analysis of a Superdex75 gel filtration of a mixture of recombinant SycO (15.9 kDa) and the monomeric marker protein carbonic anhydrase (29 kDa). The Coomassie-stained gel of consecutive fractions shows that SycO elutes before the 29 kDa marker protein. Calibration of the column revealed an apparent molecular mass of approximately 35 kDa for SycO, which is well in accordance with dimer formation. To confirm this, we performed a chemical cross-linking experiment as shown in Fig.1B. The cross-linking reagent DSP generated heat-stable coupling products corresponding to SycO dimers as resolved by SDS-PAGE. Note that the mobility of SycO in SDS-PAGE is higher than that of typical marker proteins due to its low isoelectric point of 4.3.

### Overproduction of SycO represses Yop secretion in *Y. enterocolitica*

**Figure 2:**
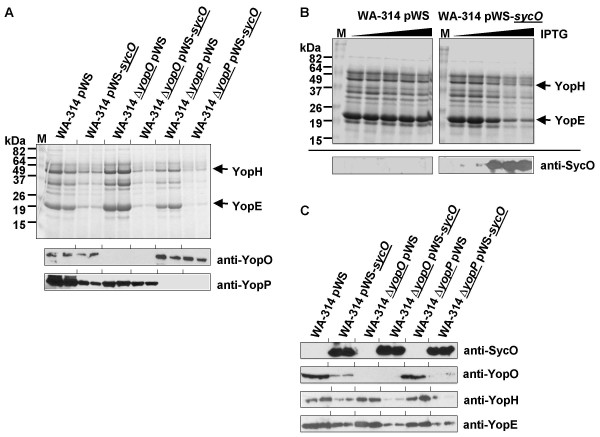
Overproduction of SycO represses secretion and expression of Yops. *Y. enterocolitica *parental strain WA-314 and the isogenic deletion mutants Δ*yopO *and Δ*yopP *were transformed with plasmids pWS-*sycO *and pWS, respectively. After culturing at 37°C in the presence of 0.2 mM CaCl_2 _for 2 h, Yop secretion was induced with EGTA. Simultaneously, the cultures were supplemented with 1 mM IPTG and grown for another 2 h. (A) TCA-precipitated culture supernatants were analyzed by Coomassie-staining of an SDS gel (upper panel), and by immunoblotting of YopO and YopP (lower panels). Two independent cultures of each strain are represented. Calibration of gel loads was obtained by measurement of the optical density of the cultures. (B) Concentration-dependency of the SycO overproduction phenotype. Yop secretion was induced in *Y. enterocolitica *WA-314 harboring plasmids pWS-*sycO *and pWS, respectively, and simultaneously SycO overproduction was induced by addition of IPTG in increasing amounts (indicated by the filled triangles). The IPTG concentrations were 0, 0.001, 0.01, 0.1, and 1 mM, respectively. The upper panel shows a Coomassie-stained SDS gel after separation of TCA-precipitated supernatants. The lower panel shows an immunoblot of bacterial pellets to detect the expression of SycO. (C) Cell lysates corresponding to the secretion analysis presented in (A) were analyzed by immunoblotting of SycO, YopO, YopH, and YopE.

It was described that overproduction of the regulatory T3SS chaperone SycH in *Yersinia *influences regulation of Yop secretion and expression [25,26]. In order to test SycO for possible regulatory functions, we overproduced SycO in *Y. enterocolitica *WA-314 and in the isogenic Δ*yopO *and Δ*yopP *mutant strains [27]. In all three strains, the overproduction of SycO reduced the level of secreted Yops significantly (Fig. 2A). An IPTG titration series is shown in Fig. 2B illustrating the correlation between an increase of SycO production and a decrease of Yop secretion. As an exception, the level of secreted YopO remained stable upon SycO overproduction in the parental strain and in the Δ*yopP *mutant strain as revealed by Western blotting (Fig. 2A).

Strikingly, analysis of the pellet fractions revealed a decrease of all Yops including YopO within the cells overproducing SycO (Fig. 2C). It was recently demonstrated, that secretion of YopO into the culture supernatant does not strictly rely on SycO [20,23]. Thus, excess of SycO could increase the secretion efficiency of YopO. The finding that the amount of secreted YopO was constant despite of reduced intracellular levels suggests that YopO secretion efficiency was increased by excess of SycO.

On closer inspection of YopP secretion, it became evident that deletion of *yopO *generated a polar effect resulting in reduced YopP secretion (Fig. 2A). Interestingly, SycO overproduction did not cause as pronounced a reduction of YopP secretion in the *yopO *mutant as in the parental strain. This observation might point to an interrelation between YopO/SycO and YopP and hence to an explanation for the operon structure.

Taken together, these results suggest that SycO overproduction interferes with the T3SS regulatory network. Strikingly, this effect is neither mediated by an interaction of SycO with its effector YopO nor by an interaction with YopP which is also encoded within the same operon.

**Figure 3:**
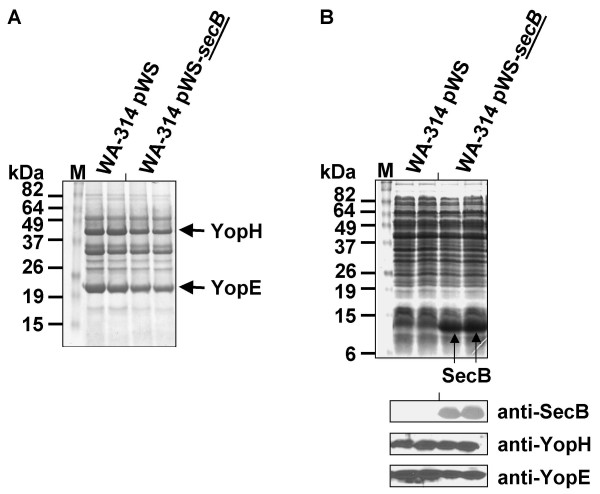
Overproduction of SecB does not cause significant effects on secretion and expression of Yops. Analogous to the experimental setup described in legend to Fig. 2, SecB was overproduced. (A) Coomassie-stained SDS-PAGE after loading of TCA-precipitated fractions of the culture supernatant. (B) Analysis of the corresponding cell lysates; Coomassie-stained gel (upper panel) and corresponding immunoblots to detect SecB, YopH, and YopE (lower panels).

To test a possible unspecific effect of SycO overproduction, we searched for a protein with similar functions and properties but unrelated to the T3SS to overproduce it as a control. We chose SecB, the chaperone of the Sec transport pathway, which exhibits similar properties compared to SycO (SecB: 17.3 kDa, pI 3.91; SycO: 15.9 kDa, pI 4.32). Overproduction of SecB in the same strains did not cause any significant effect on secretion and expression of Yops (Fig. 3), suggesting a specific effect of SycO towards the T3SS.

As a further control of specificity, we analyzed the influence of SycO overproduction on the Sec-pathway by monitoring beta-lactamase-mediated resistance to carbenicillin. We found no influence of SycO overproduction on the level of carbenicillin resistance even at concentrations above 1 mg/ml of carbenicillin (data not shown).

**Figure 4:**
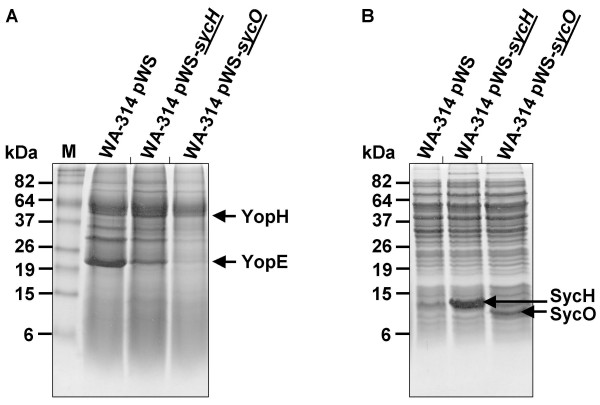
The SycH overproduction phenotype differs significantly from that of SycO. Analogous to the experimental setup described in legend to Fig. 2, SycH and SycO were overproduced in yersiniae. (A) Coomassie-stained SDS-PAGE after loading of TCA-precipitated fractions of the culture supernatant. (B) Coomassie-stained gel of the corresponding cell lysates.

Finally, we compared the effect of SycO overproduction to that of SycH overproduction (Fig. 4). Even though the overproduction of SycH was very massive compared to SycO (Fig. 4B) no similar effect on secretion of Yops was observed (Fig. 4A). In conclusion, these findings suggest that the SycO overexpression phenotype is due to a specific interaction of SycO with some component of the T3SS or one involved in the T3SS regulatory network.

### Differential effect of SycO overproduction on accumulation of Yops in the *Yersinia *cytosol in the presence of calcium ions

**Figure 5:**
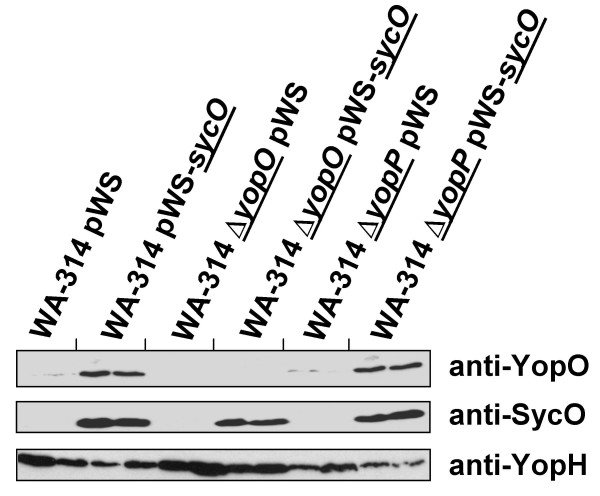
Differential effect of SycO overproduction on cytosolic Yop levels in the presence of calcium ions. Overproduction of SycO was analyzed in *Y. enterocolitica *parental strain WA-314 and the isogenic deletion mutants Δ*yopO *and Δ*yopP *cultured at 37°C in the presence of 5 mM CaCl_2_. After 2 h 1 mM IPTG was added and cultivation was continued for another 2 h. Pellet fractions were analyzed by immunoblotting with sera raised aginst YopO, SycO and YopH.

We further analyzed the effect of SycO overproduction in the presence of 5 mM CaCl_2 _at 37°C to detect a possible calcium-blind phenotype. We observed that none of the strains secreted Yops in the presence of calcium (data not shown). Interestingly, SycO overproduction increased the cytosolic YopO levels (Fig. 5). An excess of SycO possibly stabilizes YopO and thereby increases its half-life when no secretion takes place. In comparison, YopH levels were not significantly affected upon overproduction of SycO (Fig. 5). Consequently, SycO overproduction interferes with Yop expression only under conditions of Yop secretion.

### SycO interacts with the T3SS regulatory protein YscM1

**Figure 6:**
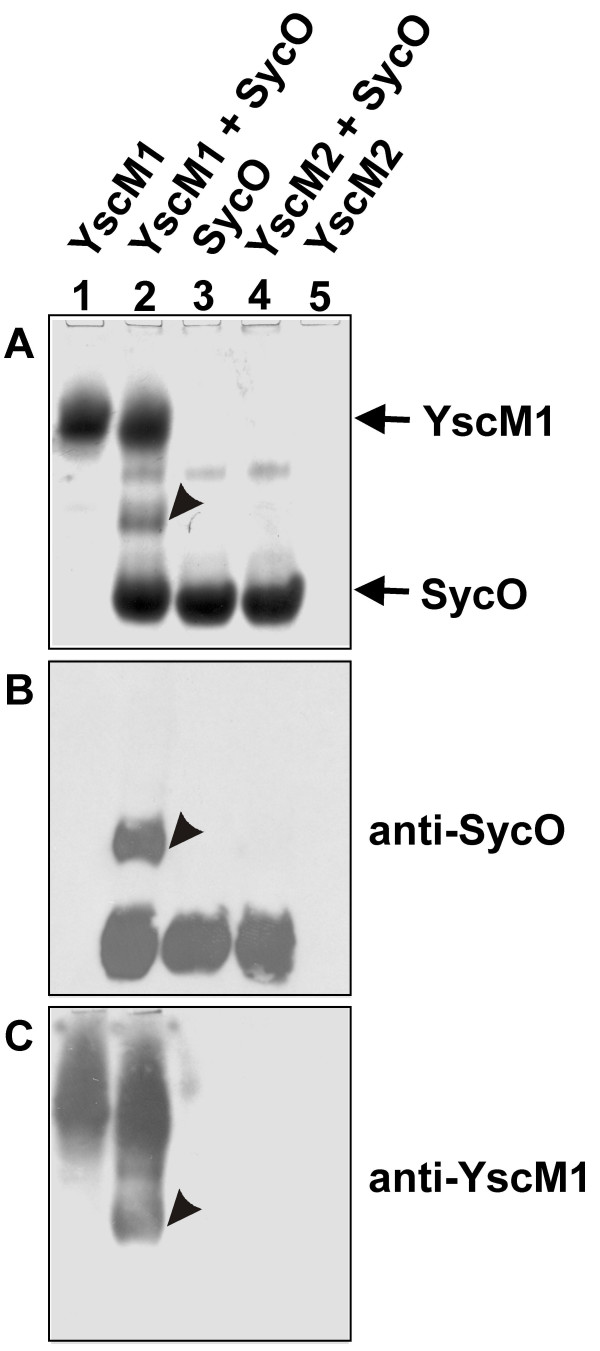
Native gel electrophoresis reveals an interaction between SycO and YscM1. Recombinant proteins as indicated were loaded on Tris-buffered native gels. The gels were Coomassie-stained (A) or alternatively electro-blotted and incubated with antisera raised against SycO (B), and YscM1 (C), respectively. The amount of SycO (20 μg) was approximately equimolar to that of YscM1 (15 μg) and YscM2 (15 μg). SycO/YscM1 complex is indicated by arrowheads. YscM2 does not enter the native gel (pH 8) due to its high isoelectric point (pI 9.79).

Several *Yersinia *T3SS chaperones like SycD, SycE, and SycH are known to be involved in regulation of Yop expression [26,28,29]. Interestingly, these chaperones interact with the negative regulators of Yop expression, YscM1 (orthologous to LcrQ of *Y. pestis *and *Y. pseudotuberculosis*) and YscM2, respectively [25,30,31]. YscM2 is only present in *Y. enterocolitica *and paralogous to YscM1, sharing 57% identical residues [32]. Since our results suggest a regulatory function of SycO, we speculated that SycO might also interact with YscM1 and YscM2. To check these interactions, we performed native gel electrophoresis as shown in Fig. 6. A representative Coomassie-stained native gel (Fig. 6, panel A) shows that loading a mixture of the recombinant proteins YscM1 and SycO results in appearance of an additional band (lane 2). This band does not appear when loading either YscM1 (lane 1) or SycO (lane 3) alone. Immunoblots developed with antisera raised against SycO (panel B) and YscM1 (panel C) revealed that this band represents a complex composed of YscM1 and SycO. In contrast, no interaction between YscM2 and SycO was found (lane 4). Note that due to the high isoelectric point of YscM2 (pI 9.79) this protein does not enter the native gel with a pH of 8 [31].

Cross-linking experiments were performed to verify the YscM1/SycO interaction. However, the data did not help to interpret the results from native gel electrophoresis since YscM1 and SycO are very similar in size and both form homodimers ([33], Fig. 1). As a consequence, the major cross-linking products could not be unambiguously assigned to heterocomplexes (data not shown).

### Overproduction of SycO in the *yscM1 *and *yscM2 *deletion mutants

**Figure 7:**
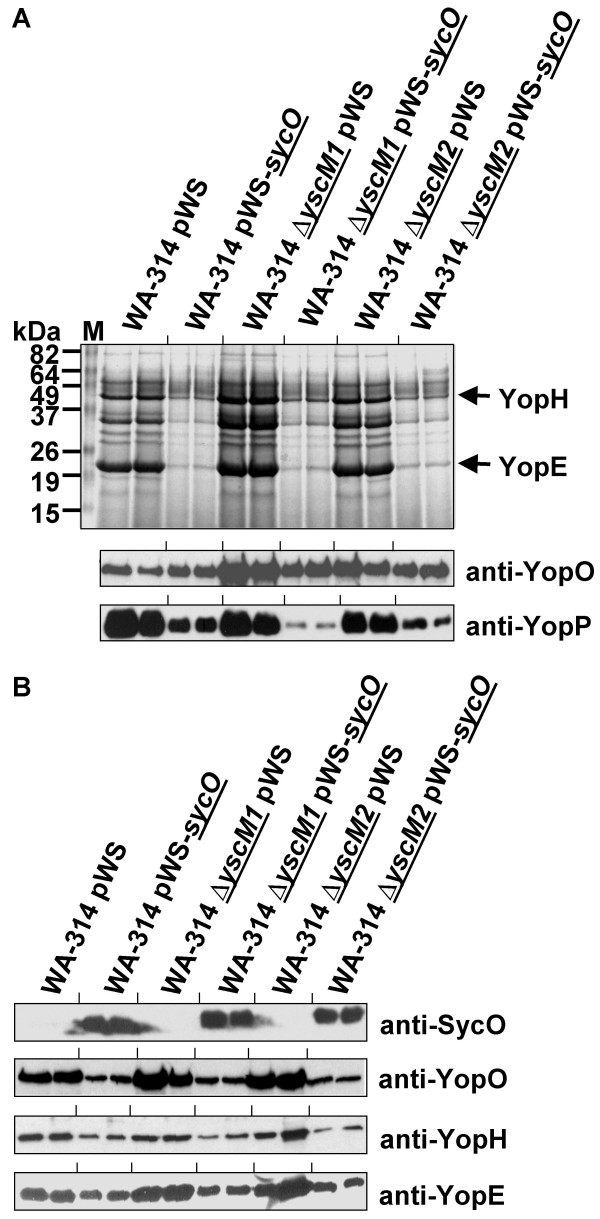
Repression of Yop secretion by SycO overproduction is neither mediated by YscM1 nor by YscM2. Overproduction of SycO was analyzed in *Y. enterocolitica *parental strain WA-314 and the isogenic deletion mutants Δ*yscM1 *and Δ*yscM2 *as described in legend to Fig. 2. Analysis of the culture supernatant is represented in (A) and the cell lysates are shown in (B).

**Figure 8:**
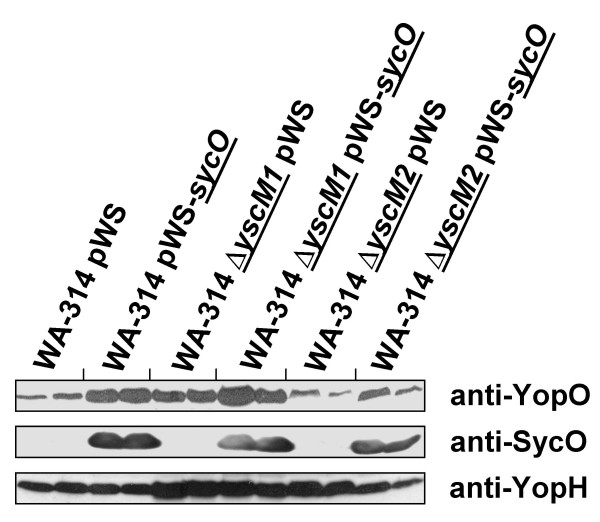
SycO overproduction in the mutant strains Δ*yscM1 *and Δ*yscM2 *in the presence of calcium ions. Overproduction of SycO was analyzed in *Y. enterocolitica *parental strain WA-314 and the isogenic deletion mutants Δ*yscM1 *and Δ*yscM2 *cultured at 37°C in the presence of 5 mM CaCl_2_. After 2 h 1 mM IPTG was added and cultivation was continued for another 2 h. Pellet fractions were analyzed by immunoblotting with sera raised aginst YopO, SycO and YopH.

We speculated that the effects of SycO overproduction described above could be mediated by the regulatory proteins YscM1 and YscM2. To find out whether such a connection exists, we generated deletion mutants of *yscM1 *and *yscM2*, respectively, to use them as background for SycO overproduction. Deletions of *yscM1 *and *yscM2 *were generated by the Red recombinase based method of Datsenko and Wanner [34] that was adapted to *Y. enterocolitica *[35]. In accordance with Stainier *et al*. [32], we found that neither the Δ*yscM1 *nor the Δ*yscM2 *mutant exhibited a phenotype with respect to Yop expression and secretion (Fig. 7). Overall, overproduction of SycO in both mutant strains caused a decrease of Yop secretion (Fig. 7A) and expression (Fig. 7B) similar to what was observed for the parental strain. However, two reproducible phenomena related to the *yscM1 *deletion strain could be observed. First, SycO overproduction caused a decrease of YopO secretion (Fig. 7A). In the presence of extracellular calcium, the increase of YopO levels was less pronounced in the Δ*yscM1 *and also in the Δ*yscM2 *strain compared to the parental strain (Fig. 8). Second, decrease of YopP secretion as a consequence of SycO overproduction was more pronounced in the Δ*yscM1 *strain compared to any other strain (Fig. 7A and Fig. 2A for comparison).

In summary, these results show that neither YscM1 nor YscM2 is required to establish the SycO-mediated shutdown of Yop expression and secretion. However, the *yscM1 *deletion mutant exhibits a phenotype suggesting that SycO and YscM1 act together in the regulation of YopO and YopP.

## Discussion

In this study, we demonstrate that *Y. enterocolitica *SycO behaves as a homodimer in solution. This is in full agreement with a recent study from Letzelter *et al*. [20] who also applied gel filtration analysis and cross-linking techniques with the same outcome. Homodimer formation is typical for T3SS chaperones of effectors, and SycO dimerization is thus plausible in the light of the recent discovery that SycO serves as a chaperone for YopO [20]. Now, that we have established a protocol for the large-scale production of native SycO we aim at generating high-resolution structural data to characterize SycO on the molecular level.

We have further presented evidence that the functions of SycO are not restricted to chaperoning YopO. We could show that overproduction of SycO causes an apparent repression of Yop secretion and expression. Two principle explanations for this phenomenon seem plausible. On the one hand, SycO could interfere with the regulatory network involved in Yop expression, thereby causing down-regulation of Yop expression and consequently reducing the amount of secreted Yops. On the other hand, SycO could interfere with some component of the T3SS transport machinery. In particular, T3SS chaperones are capable of interacting with the specific ATPase of the transport machinery [9] irrespective of effector-binding to the chaperones. Thus, abundant SycO could compete with chaperone/Yop complexes and thereby reduce the efficiency of Yop secretion. Since a shutdown of secretion typically results in feedback inhibition of Yop expression, this scenario could explain the phenotype equally well. This latter hypothesis is challenged by the observation that overproduction of SycH in *Y. pestis *causes a slight increase of Yop expression and secretion as well as a relaxation of the Calcium-dependent control of Yop secretion [26]. Similarly, moderate overproduction of SycH induces Yop production in *Y. enterocolitica *and *Y. pseudotuberculosis *[25]. Even the dramatically elevated SycH levels that we applied could not provoke an effect comparable to that of SycO. Therefore, an interference of SycO with the Yop regulatory network is a more likely explanation. Since YopO and YopP proved to be dispensable for mediating the effect caused by SycO overproduction, we speculated on a role of the Yop regulatory proteins YscM1 and YscM2 in this context. Clearly, neither YscM1 nor YscM2 was required to establish the SycO phenotype. Presumably, at least one further interaction partner of SycO has to be identified. This will help to understand whether SycO overexpression acts at the level of transcription or translation.

Nevertheless, we could demonstrate an interaction between YscM1 and SycO *in vitro*. In support of such an interaction, overproduction of SycO in the *yscM1 *deletion strain influenced YopO and YopP secretion and intracellular accumulation of YopO. The YscM proteins are involved in the post-transcriptional regulation of Yop expression. In addition, they are T3S substrates themselves and chaperoned by SycH, another actor in the Yop expression network [25,36,37]. Recently, binding of SycE to YscM1 and YscM2, and binding of SycD to YscM2 was demonstrated [30,31]. What could be the meaning of this YscM promiscuity? An unspecific interaction of both YscM1 and YscM2 with the acidic T3SS chaperones seems unlikely, given that the isoelectric point of YscM1 is 6.05 whereas the pI of YscM2 is 9.79. We propose that the YscM proteins function as an interface, integrating information on whether T3SS chaperones are loaded with Yops, and transducing these signals into repression or derepression of Yop translation.

## Conclusion

This study supports the recently established view that SycO is a typical T3SS chaperone of class I that increases solubility and secretion efficiency of the effector YopO [20]. Beyond that, our results suggest that SycO is part of the T3SS regulatory network. This finding highlights the need for a complete T3SS interactome to understand the complex T3SS machinery.

## Methods

### DNA manipulation

*Yersinia enterocolitica *WA-314 [24]*sycO *was amplified by PCR using primers 5'-AAAAAACATATGATTAACTCAACCTTTACTGAGC-3' and 5'-ACTCGAGGTCGACTCAATAACCGATTGAGTAGATTGAGTAAG-3', thereby introducing NdeI and SalI restriction sites, respectively. The NdeI-SalI cleaved PCR product was introduced into expression vector pWS [18] resulting in plasmid pWS-*sycO*. *Y. enterocolitica *WA-314 *secB *was cloned into pWS after generation of a PCR product using primers 5'-CATATGTCAGAACAAAACAACACCGAG-3' and 5'-GTCGACTCAATGGTGATGGTGATGGTGGGCATCCTGACGTTGTTCAGC-3', and subcloning of the PCR product into pGEM-T (Promega). The resulting plasmid was termed pWS-*secB*. Construction of plasmid pWS-*sycH *was conducted in an analogous manner using primers 5'-CATATGCGCACTTACAGTTCATTAC-3' and 5'-GTCGACTTAAACCAGTAAATGAGATGATG-3'. Deletion of *yscM1 *and *yscM2*, respectively, in *Y. enterocolitica *WA-314 was introduced applying the phage lambda Red recombinase technique as previously established for *Y. enterocolitica *[35]. The kanamycin gene of plasmid pACYC177 (New England Biolabs) was amplified with 50-nucleotide-homology extensions corresponding to the flanks of the *yscM1 *coding region using primers 5'- AATAAATAACTTAGAATATCGTAGAGATAATTATAGCGACAGGAGACTCGTCACTGACACCCTCATCAGTG-3' and 5'- ATCAACCTGGGGGTATTATCGCCTCAATATACAGTAATATATTATCGCGTCAAGTCAGCGTAATGCTC-3'. Analogously, primers 5'-TGGTGGTTTAGTTTGTGTTTATTTTAAAATATACATGACATATCGACGTTTCACTGACACCCTCATCAGTG-3' and 5'- TGAAAAATTGAATTTTTAGTTTTAAGGTAACTTATCATTAAGTTGTGATACGTCAAGTTGTGATACGTCAAGTCAGCGTAATGCTC-3' were used in the case of *yscM2*. Construction of *yopO *and *yopP *deletion mutants was described previously [27].

### Culturing of yersiniae

Induction of the T3SS of *Y. enterocolitica *WA-314 was accomplished essentially as described [38]. In brief, yersiniae overnight cultures grown at 27°C in brain heart infusion broth (BHI; Difco) were diluted 1:20 in BHI supplemented with 0.2 mM CaCl_2 _and cultured for 2 h at 37°C. Then, EGTA was added to a final concentration of 5 mM and MgCl_2 _to a final concentration of 10 mM for induction of Yop secretion. Alternatively, for repression of Yop secretion yersiniae were cultivated for 4 h at 37°C in BHI supplemented with 5 mM CaCl_2_. Overproduction of SycO and SecB was induced by addition of 1 mM IPTG at the time of EGTA supplementation.

### Expression and purification of recombinant SycO

The strategy for production of recombinant SycO was essentially as described for SycT [18]. Plasmid pWS-*sycO *was transformed into BL21 (DE3) pLysS (Stratagene). A 100 ml overnight culture was grown in LB medium supplemented with 1% glucose at 37°C and diluted 1:50 in 5 liter LB medium. The culture was further grown in a BIOSTAT B fermenter (B.Braun Biotech International) at 37°C under conditions of constant aeration (10 l/min), pH (7.2) and stirring (200 rpm). At an OD_600 _of 0.7 the temperature was decreased to 27°C and subsequently SycO expression was induced by adding 0.2 mM IPTG. Bacteria were cultivated for another 18 h, harvested by centrifugation and frozen at -20°C. The bacterial pellet from 1.25 liter of culture was resuspended in 80 ml of Buffer A (50 mM Tris-HCl pH7.6, 100 mM NaCl, 2 mM DTT) supplemented with 4 mM PMSF and 250 U Benzonase (Merck Biosciences). The sample was passed through a French press three times (1000 psi) and centrifuged at 20,000 g for 40 min. The supernatant was adjusted to 20% (NH_4_)_2_SO_4 _and gently stirred for 2 h at 4°C. The SycO precipitate was obtained by centrifugation (20,000 g, 4°C), washed with precipitation buffer and resuspended in buffer A. The sample was then loaded on a MonoQ column (HiTrap Q FF, 5 ml; Amersham Biosciences) equilibrated with buffer A. The column was washed with 10 ml of buffer A, then elution was conducted by applying a 100 ml linear gradient of 0–100% elution buffer (50 mM Tris-HCl pH 7.6, 1 M NaCl, 1 mM DTT). SycO eluted in the range of 280–370 mM NaCl.

### Chemical cross-linking

Cross-linking reagent DSP (dithiobis [succinimidylpropionate]) was purchased from Pierce. For cross-linking of SycO, 10 μl reaction volume containing 1.3 mM DSP and 3 μg/μl recombinant SycO in phosphate buffered saline (PBS) was incubated for 30 min at 22°C. The reaction was stopped by adding 0.4 μl of a 1 M Tris-HCl pH 7.5 solution and was incubated for another 15 min at 22°C. 5 μl of SDS-PAGE loading buffer containing 0.5% 2-mercaptoethanol were added and the sample was heated for 5 min at 95°C before loading.

### Native gel electrophoresis

Tris-buffered continuous native gel electrophoresis was performed as previously described [31]. Gels were stained with Coomassie Brilliant Blue or were alternatively electro-blotted.

### Analytical gel filtration chromatography

Analytical gel filtration was performed with a SMART system (Amersham Biosciences) as described [33]. Recombinant SycO (50 μg) and the monomeric 29 kDa protein carbonic anhydrase (50 μg) were mixed in a volume of 50 μl of PBS and loaded on a Superdex 75 PC 3.2/30 column (Amersham Biosciences) equilibrated with PBS. Fractions of 50 μl were collected and analyzed by SDS-PAGE. Marker proteins for column calibration were purchased from Sigma (bovine serum albumine, 67 kDa; carbonic anhydrase, 29 kDa; cytochrome *c*, 12.4 kDa).

## Authors' contributions

SD, AS, JH, and GW conceived of the study. SD carried out analytical gel filtrations, expression and secretion analyses, and edited the manuscript. AS performed native gel electrophoresis, chemical cross-linking experiments, and edited the manuscript. SR generated recombinant plasmids and performed expression and secretion analyses. KT generated deletion mutants and edited the manuscript. JH supervised the work, and edited the manuscript. GW coached the lab staff, and wrote the draft of the manuscript. All authors read and approved the final manuscript.
